# Progress and challenges in designing dynamic *in vitro* gastric models to study food digestion

**DOI:** 10.3389/fnut.2024.1399534

**Published:** 2024-06-05

**Authors:** R. Paul Singh

**Affiliations:** Distinguished Professor Emeritus of Food Engineering, University of California, Davis, Davis, CA, United States

**Keywords:** food digestion, *in vitro* models, gastric digestion, food breakdown, gastric simulator

## Abstract

Understanding the mechanisms involved in food breakdown in the human gastrointestinal (GI) tract is essential in food digestion research. Research to study food digestion in the human GI tract requires *in vivo* and *in vitro* approaches. *In vivo* methods involving human or animal subjects are often cost-prohibitive and raise ethical concerns. For these reasons, *in vitro* approaches are becoming more common. Several dynamic *in vitro* models that mimic one or more components of the GI tract have been developed at various research institutions and by commercial companies. While there is evidence of considerable novelty and innovation in the design of these models, there are many differences among them in how the mechanical breakdown of solid foods is accomplished. In some systems, modulating water pressure is used to achieve peristaltic contractions of the gastric antrum, whereas, in other models, the flexible walls of a gastric chamber are compressed by the movement of rollers or clamps outside the walls of the test chamber. Although much progress has been made in standardizing the biochemical environment appropriate to the food digestion process, there is a lack of standard protocols to measure mechanical forces that result in the breakdown of solid foods. Similarly, no standardized methods are available to evaluate the results obtained from *in vitro* trials for validation purposes. Due to the large variability in the design features of *in vitro* models used for food digestion studies, developing consensus-based standards for the mechanical aspects of food breakdown is needed.

## Introduction

The importance of improving the understanding of food digestion has promoted the need for dynamic *in vitro* models of the gastrointestinal (GI) tract. Within the GI tract, solid foods undergo size reduction to allow the release of nutrients that may ultimately pass through the intestinal walls. Experimental studies on food digestion involving human subjects are often cost-prohibitive and involve ethical and operational barriers. Therefore, *in vitro* models, mimicking the human digestive tract, are necessary to advance the field. Since the early 2000s, considerable progress has occurred in developing *in vitro* models of various parts of the human GI tract for food and pharmaceutical applications (1).

Digestion of solid foods in the GI tract is influenced by the surrounding biochemical media and the mechanical forces created within the tract, such as chewing and mastication in the mouth and peristaltic contractions in the antrum stomach. Biochemical digestion involves exposure of the digesting food to various chemicals and enzymes secreted inside the GI tract. The recipes and protocols for creating biochemical environment in an *in vitro* model to simulate human *in vivo* conditions of the GI tract have been recently standardized (2). However, accurately mimicking the physiologically derived mechanical forces acting on solid foods during digestion remains challenging. While some design features of different *in vitro* models are common, significant differences exist in how the mechanical forces are applied to the digesting food. Almost every *in vitro* model has its own unique mechanism to create mechanical forces. Furthermore, the methods used to validate experimental results vary among researchers. Some researchers measure magnitude of mechanical forces, others rely on indirect procedures involving breakdown of analog materials such as agar gel beads. This paper reviews the design characteristics of selected *in vitro* models with a particular reference to how the mechanical breakdown of digesting foods occurring in the gastric component of the GI tract is accomplished and validated. The need to standardize the methods used to measure and validate mechanical forces in an *in vitro* model will be presented.

## Selected dynamic *in vitro* models to study food digestion

**FIGURE 1:**
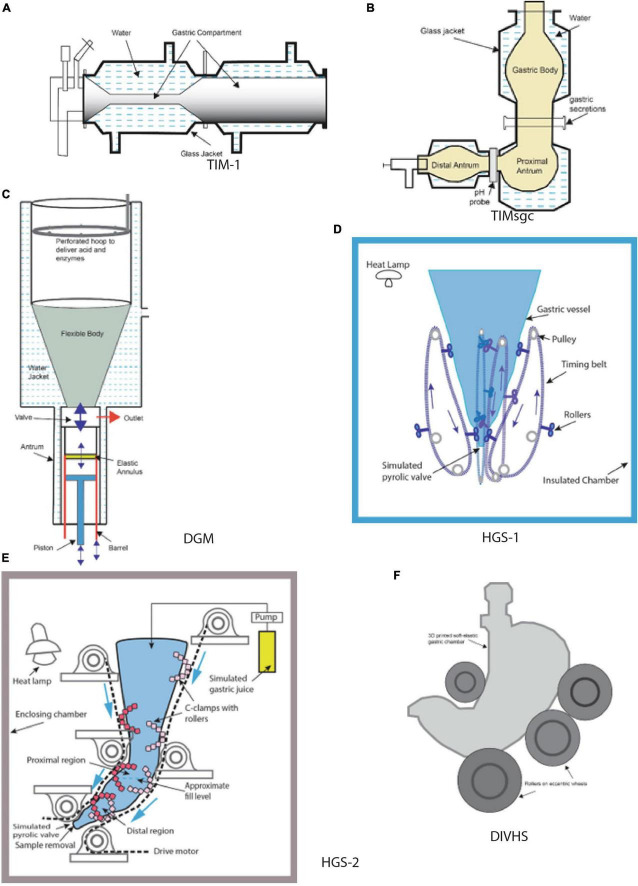
Schematic diagrams of selected gastric *in vitro* systems **(A)** TIM-1, **(B)** tiny-TIMsgc, **(C)** DGM, **(D)** HGS-1, **(E)** HGS-2, **(F)** DIVHS. **(A)** Adapted from (32), **(B)** adapted from (8), **(C)** adapted from (9).

This section presents the design characteristics of selected dynamic *in vitro* models of the upper GI tract. These models are selected based on their wide use in food digestion studies or innovative design features to mimic *in vivo* conditions. Schematic diagrams of some models are shown in Figure 1, and some key features are presented in Table 1. For additional details about the *in vitro* models presented in this paper, the reader is referred to review papers (3–6).

**TABLE 1:** Physical mechanisms used for solid food breakdown in gastric compartments and selected design characteristics of dynamic *in vitro* models.

*In vitro* model	Mechanism used for peristaltic contractions in the gastric compartment	Approximate volumetric capacity (mL)	Inside gastric surface
TIM-1	Modulating water pressure around flexible wall compartments	300	Smooth
tiny-TIMsgc	Modulating water pressure around flexible wall compartments	300	Smooth
DGM	Mechanical movement of an elastic annulus in the antrum	800	Smooth
HGS-1	Wheels on a belt moving on flexible walls of the gastric chamber	5700	Smooth
HGS-2	C-clamps with rollers moving on flexible walls of the gastric chamber	900–1000	Smooth
DIVHS	Rollers on eccentric wheels create peristalsis contraction of flexible walls	400	Simulated gastric interior wall
DIDGI	A propeller stirs the gastric content	940	Smooth
ESIN	A shaft stirrer is used with adjustable rotors	Not available	Smooth
GSM	Pneumatically driven syringes compress the gastric wall	600	Smooth
IMGS	Multiple pistons located on the sides of the stomach chamber are used for compressing the gastric chamber	900	Smooth

**TIM** (TNO Intestinal Model) dynamic *in vitro* models **(TIM-1, TIM-2, tiny-TIMsg)** were developed at TNO Triskelion, Zeist, the Netherlands, and commercialized at InnoGI Technologies (formerly The TIM Company). **TIM-1** is a multi-component model comprising the stomach, duodenum, jejunum, and ileum (1, 7), **tiny-TIMsg** is a 2-component system comprising a stomach and a single component for the small intestine, and **TIM-2** comprises four independent large intestinal compartments. The stomach component in TIM-1 is represented by a horizontally oriented flexible tube placed in a transparent rigid cylinder (Figure 1). Water at 37°C is circulated inside the annular space between the flexible wall of the tube and the outer rigid cylinder to provide modulated contractions of tube contents. The pressure forces created inside the tubular stomach region have been validated with *in vivo* human data. The operating protocols allow control of temperature and pH. The release rate of secretions of simulated gastric juice is adjustable based on the test food. The operating controls allow the creation of conditions to mimic the stomach functions of neonates, infants, toddlers, adults, and the elderly. The large intestine compartment in TIM-2 model allows inoculation of its inside walls with human fecal samples. Among various dynamic *in vitro* systems, TIM models are notable for most pharmaceutical and food applications. Numerous papers have been published on food digestion using the TIM models (1). A relatively newer *in vitro* model in the TIM series is tiny-TIMsg (smartifcialgut) (8). In this model, the gastric component is represented by three parts: the first two (gastric body and proximal antrum) are vertically oriented, and the other one (distal antrum) is horizontal to mimic the J-shape of the human stomach (Figure 1). Test samples with salivary secretions are introduced in the gastric body, and the simulated gastric juice is injected between the gastric body and the proximal antrum. The connection between the proximal and the distal antrum contains pH electrodes. Similar to the other TIM models, the digesta moves inside flexible tubes contained in a transparent rigid jacket. The water flow in the annular space helps create modulated contractions and is computer controlled to achieve desired motility of the digesta. Sensors are located on the flexible walls to measure the pressure. During digestion of different foods, the pressures in the gastric region are reported to be between 2 and 18 mm Hg (8). The TIM models are now coalesced under a new company, InnoGI Technologies, and are part of the *SurroGut* platform.

**DGM** (Dynamic Gastric Model) was developed at the Institute of Food Research, Norwich, UK. It was one of the early *in vitro* models incorporating biochemical and mechanical aspects of gastric digestion (9). The fundus part of the stomach is a flexible wall funnel-shaped vessel surrounded by a water jacket. The water flow in the jacket is regulated to provide rhythmic movements of the flexible wall of the vessel (Figure 1). The antrum region comprises a rigid barrel containing a piston. The food bolus transferred from the fundus region by the movement of the piston undergoes breakdown due to shearing action created by the movement of an elastic annulus that moves up and down 3 times per minute. The displacement rate of the annulus is based on data obtained from *in vivo* trials. The outlet valve from the barrel controls the exit of the digested sample at timed intervals. In the DGM, pH electrodes regulate the introduction of gastric secretions. The forces created in the antrum region were validated by studying the breakage of agar gel beads (spherical, 1.27 cm diameter of different fracture strengths) compared to *in vivo* trials conducted with human subjects (10). In addition to validating the results obtained from DGM, these authors also noted that when a USP (United States Pharmacopeia) Dissolution Apparatus II system was used in a similar trial, the agar gel beads did not disintegrate but underwent only surface erosion, emphasizing the shortcomings of the USP system that uses a paddle turning inside a rigid vessel to achieve the breakdown of solid materials. DGM has been widely used in food and pharmaceutical research.

**HGS** (Human Gastric Simulator), version 1, was designed and fabricated at the University of California, Davis (11). The model consists of a vertically oriented stomach chamber shaped like a cylinder and tapering to the bottom (Figure 1). The flexible walls are made of latex rubber. The bottom of the chamber empties into a plastic tube connected to a peristaltic pump to empty the digesta. A polyester mesh bag with a pore size of 1.5 mm is placed inside the chamber to allow passage of digested content of size less than 2 mm. Simulated gastric fluids are introduced at different locations along the inside wall of the chamber. Peristaltic contractions are created by moving custom-built rollers connected to belts operated with pulleys along the four opposing sides of the chamber. The distance between the opposing rollers decreases as they move down, increasing the contraction in the distal antrum region. The contraction forces created in the model were measured using a thin-walled rubber bulb connected to a pressure manometer. The maximum stress in the distal antrum region was measured to be 6,738 N/m^2^ when the gap between the opposing rollers was 12 mm (11). The design of this model has been replicated into multiple units used in research on gastric digestion at the Riddet Institute (Massey University, Palmerston North, New Zealand).

**HGS** (version 2), the second generation of the original simulator, was designed at the University of California, Davis, to create a J-shaped chamber with circumferential peristaltic contractions. The contractions are obtained using C-clamps containing custom-designed Teflon rollers (12). The separation between the opposing C-clamps along the chamber’s walls is controlled to obtain the contraction forces inside the distal antrum (Figure 1). Using a rubber bulb attached to a pressure manometer, the maximum contraction force was measured in the gastric chamber as 5.9 ± 0.3 N or normalized by the sectional area of the rubber bulb to be 8347 ± 424 N/m^2^ (13). The *in vitro* model was recently validated using data from the digestion of starch-based foods obtained from *in vivo* trials conducted with growing pigs (14). Both versions of HGS have been used for numerous food digestion studies in multiple labs for the past 15 years (15–18).

**DIDGI**^®^ (Digesteur dynamique gastrointestinal) is a two-component system developed at INRA, France, representing gastric and intestinal regions. Components are made of transparent materials to allow visual observations during a digestion experiment. The stomach is represented by a rigid vessel containing a stirrer. Custom computer software controls the operating parameters, such as temperature, transit times in the gastric and intestinal regions, the addition of digestive secretions, and pH in the two regions. DIDGI^®^ has been used for digestion studies of infant formula, human milk, and various bovine milk products including cheese and skim milk. The *in vitro* system has been validated by digesting infant formula and comparing milk proteolysis in digestion studies with piglets (19). Validation studies have compared the kinetics of casein and beta-lactoglobulins evolution during digestion. With its simple design, the apparatus is robust; however, using a stirrer to accomplish mixing and breakdown fails to mimic the dynamic forces associated with peristaltic contractions in a human stomach.

**ESIN** (Engineered Stomach and Small Intestinal) was developed at the University of Auvergne, Clermont-Ferrand, France. This system has six chambers, representing an inlet chamber to introduce realistic-sized food particles into the model, a mixing chamber for the test sample to mix with simulated saliva, stomach, duodenum, jejunum, and ileum. The stomach chamber contains a rigid cylinder (methacrylate) containing two pistons moving from opposing sides. The test samples are subjected to mechanical forces generated in this cylinder/piston arrangement. Electronic systems control the temperature, spatial and temporal changes of pH, the input of simulated gastric, pancreatic, and biliary secretions, transit time, and mixing of chyme. The gastric section involves segregated emptying of small-size digested particles (<2 mm) and larger-size particles using peristaltic pumps. Pumps are used to obtain desired emptying rates. Validation trials have largely focused on the digestion of pharmaceutical drugs, such as soluble paracetamol and theophylline (20).

**GSM** (Gastric Simulator Model) was developed at the University of Georgia, Athens, Georgia, USA (21). The walls of the stomach chamber are made of latex. There are well-defined regions of the stomach, specifically, cardia, fundus, proximal corpus, and distal corpus. The pyloric opening is regulated with an air/vacuum system to allow particles smaller than 1.0–2.0 mm to pass through while retaining larger particles for further breakdown. The peristalsis contractions along the flexible chamber walls are obtained using a series of syringes placed circumferentially along the wall, from the fundus down to the pylorus. A programmable logic control system operates the syringes to obtain the desired regional contractions while creating a forward flow inside the chamber. The intragastric pressure, measured using a pipette rubber bulb with a digital manometer in the antrum region, was around 55 mm Hg. Simulated gastric secretions are introduced at different locations in the corpus using a variable flow peristaltic pump. GSM has been used to measure the breakdown of cooked sausage and the results were compared with those obtained from the conventional shaking bath method (21).

**DIVHS** (Dynamic *In Vitro* Human Stomach) was developed at Soochow University, Suzhou, China (22). It is based on previous generations of models developed by the researchers and aimed at reproducing human stomach anatomy and biochemical environment. The stomach chamber and the duodenum are fabricated using 3D printing with soft-elastic silicone rubber. The gastric chamber is J-shaped, the size of an adult human stomach (Figure 1). The stomach walls are about 5 mm thick. The peristaltic contractions of the stomach and intestine are created using a series of eccentric wheels and rollers. The amplitude of waves increases toward the distal antrum, by decreasing the distance between the rollers. The mechanical stress in the antrum region was measured to be 8,920 N/m^2^. The secretions (simulated gastric juice and intestinal fluid) are delivered using peristaltic pumps. Gastric emptying of smaller particles is achieved by rotating the platform that supports the gastric model. The entire unit is placed in a controlled-temperature chamber. This *in vitro* model has been used to digest cooked rice and a mixed meal containing beef stew and orange juice (22).

**IMGS** (*In Vitro* Mechanical Gastric System) was developed at the Universidad Tecnologica Metropolitana, Santiago, Chile. The J-shaped stomach chamber is made of 1.0 mm thick latex wall formed using a polylactic acid mold fabricated with a 3D printer (23). The walls are compressed from opposing sides using four acrylic pistons located on each side. Pistons along the fundus/body region of the chamber operate with 0.3 Nm torque, whereas the other pistons along the antrum employ a torque of 0.88 Nm. A computer control system is used to operate the pistons. Constant temperature is maintained by submerging the flexible gastric chamber in a water bath. Digestion studies have included investigating the role of gastric peristalsis on the intestinal lipolysis of protein-stabilized oil-water emulsions (23).

Other notable *in vitro* models of GI tract developed for food applications use custom designs and operating protocols (24–27).

## Discussion

While considerable progress is being made in designing new *in vitro* digestion systems, several opportunities exist for improved representation of *in vivo* conditions. Only a few *in vitro* models mimic the J-shaped anatomy of the gastric chamber. Computational fluid dynamic studies have shown complex flow patterns within a J-shaped space domain when subjected to peristaltic contractions of the flexible walls (28–30). The domain shape uniquely influences the formation and location of eddies and vortical flow. Similarly, inside the J-shaped domain, there is considerable spatial variation of shear forces. Such fluid flow patterns cannot be replicated in tubular or conical-shaped domains. By fabricating the gastric chamber with 3-D printing, an accurate representation of the J-shaped anatomy of a human stomach is now possible (DIVHS, IMGS, GSM). Most of the current *in vitro* models (except DIVHS) use a smooth surface for the inside wall, whereas in the human stomach, the inside wall has numerous odd-shaped wrinkles and small indentations that may influence the surface conditions (such as friction) where solid foods rub against the inside walls during mixing and breakdown. 3-D printing allows creating more realistic surfaces for the inside wall of the gastric chamber (4).

Only a few *in vitro* models have been validated using *in vivo* trials with human or animal subjects. The high costs of *in vivo* trials with animals or human subjects often inhibit such studies. Furthermore, food products have a diverse range of material properties. Therefore, the results obtained from a digestion study of one food may not apply to another. A possible approach is to classify solid foods into broad categories based on their material properties and the rates of solid breakdown (31). Selected *in vivo* trials with foods representing such broad categories may provide useful information to develop reliable operating conditions for different *in vitro* models.

Current *in vitro* models do not incorporate the entire gastrointestinal tract. The TIM models cover most of the GI components except for oral processing. Most models focus on one or two components, and none contain a validated oral component to represent chewing, mastication, mixing multi-food components with saliva and its enzymes, and bolus formation. Simulated oral processing of a food sample fed to the *in vitro* gastric chamber must be clearly described based on the sample’s physical properties. While the design and operating features of most *in vitro* models are limited to studies of food digestion in a healthy adult, there is an increasing need for *in vitro* studies of food digestion by the elderly. As the ratio of elderly to adult population increases in many parts of the world, *in vitro* systems specifically designed to mimic the GI tract of the elderly will be required. Similarly, *in vitro* systems appropriate for infants are needed. Some models discussed in this paper (TIM and GSM) suggest modifications for this purpose.

As noted in this paper, many *in vitro* systems have been recently developed in different research laboratories and by commercial companies. In these models, the design characteristics of the GI components and their operating protocols vary significantly. Similarly, there is considerable variation in the validation methods used. Some researchers provide quantitative measures of forces or stresses generated within the system; however, the measurement protocols are not standardized. No published papers were found that present results from digesting the same food or food analog using two or more different *in vitro* models. While each model can yield data on food breakdown, the results from these models will be more reliable if a standardized operational protocol (relevant to the design features) is developed. Inter-laboratory measurements of selected digestion parameters of selected foods using different *in vitro* models would be highly desirable. Currently, there is no standardized method to measure forces developed during digestion in different regions of the GI tract. It would be highly desirable to develop standards for dynamic *in vitro* models and their operating protocols by scientific bodies such as the INFOGEST or the National Institute of Standards and Technology (US Department of Commerce, Washington, D.C).^1^ Results using a standardized procedure to measure mechanical forces will increase the credibility of the results obtained from different *in vitro* models. Future improvements in the design and operation of vitro models of the GI tract are expected to enhance our quantitative understanding of the food digestion process and its role in human health.

## Author contributions

RS: Writing – original draft, Writing – review and editing.

## Footnotes

1
https://www.nist.gov/
